# Microresonator frequency comb based high-speed transmission of intensity modulated direct detection data

**DOI:** 10.1515/nanoph-2022-0134

**Published:** 2022-06-16

**Authors:** Peng Xing, George Fengrong Chen, Hongwei Gao, Xavier Chia, Anuradha M. Agarwal, Lionel C. Kimerling, Dawn T. H. Tan

**Affiliations:** Photonics Devices and System Group, Singapore University of Technology and Design, 8 Somapah Rd, Singapore 487372, Singapore; Microphotonics Center, Massachusetts Institute of Technology, 77 Massachusetts Avenue, Cambridge, MA 02139, USA; Materials Research Laboratory, Massachusetts Institute of Technology, 77 Massachusetts Avenue, Cambridge, MA 02139, USA; Department of Materials Science and Engineering, Massachusetts Institute of Technology, 77 Massachusetts Avenue, Cambridge, MA 02139, USA; Institute of Microelectronics, A*STAR, 2 Fusionopolis Way, #08-02, Innovis Tower, Singapore 138634, Singapore

**Keywords:** high-speed data transmission, intensity modulated direct detection, Kerr frequency comb, microresonator

## Abstract

Globally, the long-haul transmission of ultra-high bandwidth data is enabled through coherent communications. Driven by the rapid pace of growth in interconnectivity over the last decade, long-haul data transmission has reached capacities on the order of tens to hundreds of terabits per second, over fiber reaches which may span thousands of kilometers. Data center communications operate in regimes featuring shorter reaches and higher cost sensitivity. While integrated microresonator frequency combs are poised to revolutionize light sources used for high-speed data transmission over fiber, recent progress has focused largely on coherent detection schemes. Furthermore, though state-of-the-art intensity modulators are advancing in speed, it has not been demonstrated in the literature if microresonator-based comb lines can accommodate higher intensity modulated direction data (IMDD) line rates in tandem with these advancements. In this manuscript, we demonstrate the use of microresonator frequency combs pumped with a single laser for the transmission of high-speed IMDD data. We demonstrate error-free transmission of 30 Gbs^−1^ per comb non-return-to-zero data over fiber lengths of 6 km, as well as bit error rates under the forward error correction limit for propagation through 20 km of optical fiber. 60 Gbs^−1^ and 42 Gbs^−1^ pulse modulation amplitude 4 (PAM4) data modulated on each frequency comb line is further quantified to have a bit error rate under the forward error correction limit for fiber reaches of up to 6 km and 20 km respectively. The results showcase CMOS-compatible microresonator frequency comb modulated using IMDD formats as a promising technology for high-speed transmission in the data center transceiver industry.

## 1 Introduction

Optical frequency combs have accelerated innovations in various fields [1–6] such as quantum and ultrafast optical information processing [7–10], microwave photonics [11, 12] and precision metrology [13–15]. In the commercially burgeoning field of silicon photonics-based data center communications, integrated optical frequency combs appear well poised to catalyze transformation. The promise of using a single laser to generate multiple wavelengths of light, each of which may serve as a vessel for data transmission is appealing both from a technological and cost standpoint [16–19]. Noteworthy applications of frequency combs for ultra-high density optical communications over fiber include tens of Tb s^−1^ in Hydex microresonator combs recently reported by Moss et al. (44.1 Tb s^−1^) [20], and silicon nitride microresonator combs (50 Tb s^−1^) [21]. Non-microresonator-based frequency combs generated through self-phase modulation in waveguides have enabled 661 Tb s^−1^ of data transmission in multi-core fiber [22]. These impressive demonstrations of ultra-high-capacity data transmission powered by frequency combs point unequivocally to their developmental trajectory and potential to revolutionize telecommunications and data communications hardware architectures. In these aforementioned demonstrations of high-capacity data transmission powered by microresonator frequency combs, soliton states such as soliton crystals and dissipative Kerr solitons (DKS) were used to drive information transmission. Soliton crystal states are created with slow scans of the pump laser wavelength to transition from a chaotic state to the crystal state, both of which possess similar intracavity optical power [1, 28, 29]. In contrast, DKS states which are characterized by the soliton steps observed during laser scans perform optimally when the soliton state is achieved while the resonator is close to thermal equilibrium [18, 30], [31], [32], [33]. Despite their promising performance demonstrated to date, integrated frequency combs for high bandwidth optical communications over fiber thus far have mostly relied on coherent modulation formats and coherent detection [20–23]. The reliance of coherent communications on digital signal processing introduces a cost, complexity and latency overhead compared to intensity modulated direct detection (IMDD), with the complexity of digital signal processing adopted scaling with the fiber reaches [34]. For these reasons, industrial standards such as Parallel Single Mode 4 (PSM4) [24], Coarse Wavelength Division Multiplexing 4 (CWDM4) [25], 100G Lambda [26] Multi-Source Agreements (MSAs) and the IEEE P802.3ba 100GBase standards [27] utilize Direct Detection. Industry roadmaps describe the use of IMDD for shorter reaches, whereas coherent detection becomes viable only at much longer reaches and data rates on the order of hundreds of Gbaud [34–36].

On the other hand, there were a handful of IMDD-compatible modulation of frequency combs demonstrated [37–43], as well as a notable recent demonstration in magnesium fluoride crystal microresonators [42]. However, these work either utilised low line rates (up to 10 Gb s^−1^) or used only short length fibers. With the advent of even higher speed modulators of up to 60 Gb s^−1^ [44], and the burgeoning size of data centers [45], the ability for frequency comb sources to support intensity modulation of higher IMDD rates and longer reaches remains unknown.

In this paper, we demonstrate the use of a CMOS-compatible, integrated silicon nitride microresonator for the transmission of 30 Gb s^−1^ Non-Return-Zero (NRZ) and 60 Gb s^−1^ Pulse Amplitude Modulation 4 levels (PAM4) IMDD data per comb line. We find that the primary comb state, also known as a Turing pattern which is easily pumped without requiring feedback or active stabilization, while also requiring a low pump power of 20 mW in our devices, was suitable for high-speed data transmission using direct detection, thus providing a promising, easily accessible, alternative means of high-speed data transmission other than soliton crystal or DKS states. In this reported regime, the comb state is sufficiently far from where the comb line output is susceptible to rapid fluctuations, such that any thermal fluctuations either from changes in intracavity power as the laser pump wavelength is tuned into the resonance or from ambient temperature will have negligible impact on the comb power stability. In several other comb states studied here, it was found that low bit error rates or open eye diagrams could not be obtained. These comb states corresponded to the regime in which the pump-resonance detuning was further decreased compared to the Turing state where the comb line amplitudes in contrast are defined by rapid fluctuations [30, 31]. Importantly, we demonstrate the use of high-speed data transmission using a CMOS-compatible, integrated frequency comb, using intensity modulated direct detection which is especially pertinent in the data center industry, as opposed to coherent detection. Open eye diagrams are obtained for data transmission over fiber lengths of up to 20 km. Bit error rates smaller than the Forward Error Correction (FEC) threshold of 5 × 10^−5^ [24, 25] are obtained for the various characterized comb lines. Modulation formats used were 10 Gb s^−1^ NRZ, 30 Gb s^−1^ NRZ, 42 Gb s^−1^ PAM4 and 60 Gb s^−1^ PAM4. These rates are on a per comb line basis, or per lambda. These results represent high-speed transmission of IMDD data at single lane data rates and fiber reaches exceeding that specified in both PSM4 and CWDM4 MSAs.

## 2 Results

**Figure 1:**
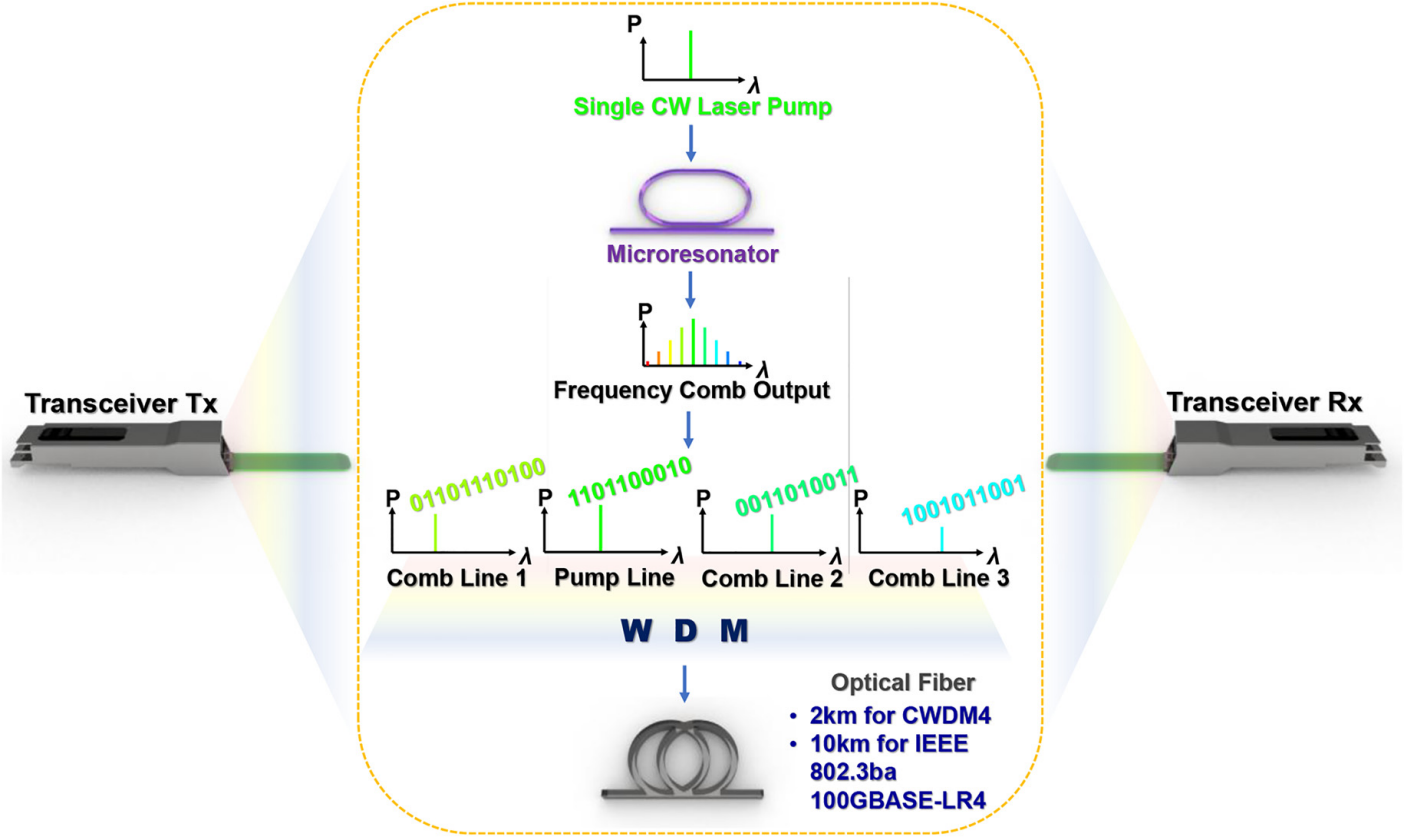
Schematic of transceiver-based data center communications. Tx: transmitter, Rx: receiver, WDM: wavelength division multiplex. The orange dotted line highlights the frequency comb-based multi-wavelength high-speed data transmission over fiber which we demonstrate in this paper.

In conventional WDM-based transceivers, each data channel is supported by a single CW laser. However, a microresonator frequency comb-based source in this work allows a single laser to generate and carry data at multiple wavelengths of light, importantly at fiber reaches and line rates exceeding that used in commercial products. Figure 1 shows a schematic of transceiver-based data center communications which can be implemented with the integrated silicon nitride microresonator frequency comb. At the transceiver transmitter (Tx) side, a single continuous-wave (CW) laser pumps a microresonator frequency comb, generating multiple wavelengths of light. Data is modulated onto each comb line prior to being wavelength division multiplexed (WDM). The multiplexed data is then transmitted through optical fiber. Examples of fiber reaches for commercial transceiver products are shown in the figure. At the end of the optical fiber at the transceiver receiver (Rx) side, the data is wavelength demultiplexed prior to detection by photoreceivers.

**Figure 2:**
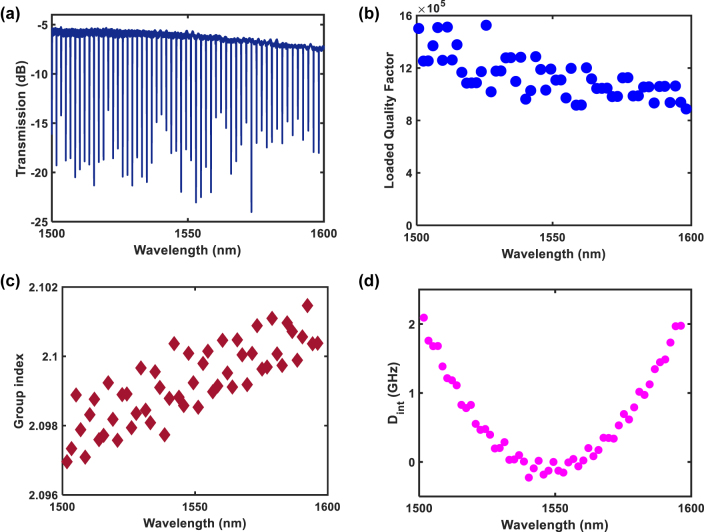
Silicon nitride microresonator optical properties. Experimentally measured (a) transmission spectrum, (b) quality factor and (c) group index of the microresonator. (d) The extracted dispersion, *D*_int_ of the microresonator.

Our Silicon Nitride (SiN) Microresonator possesses a radius of 100 μm. The cross-section of the SiN core is 800 nm (height) by 1.5 μm (width), surrounded by SiO_2_ over- and under-cladding. We first characterize the optical properties of the SiN microresonator. Figure 2(a) and (b) show the measured transmission spectrum of the microresonator and quality factor as a function of wavelength. The maximum loaded quality factor of 1.5 × 10^6^ occurs at a wavelength of 1500 nm. We note that close to 1550 nm, the wavelength at which we pump the frequency comb, the loaded and intrinsic quality factors are 1.2 × 10^6^ and 1.9 × 10^6^ respectively. The measured group index as a function of wavelength is shown in Figure 2(c). The measured group index is used to extract the microresonator dispersion as shown in Figure 2(d).

**Figure 3:**
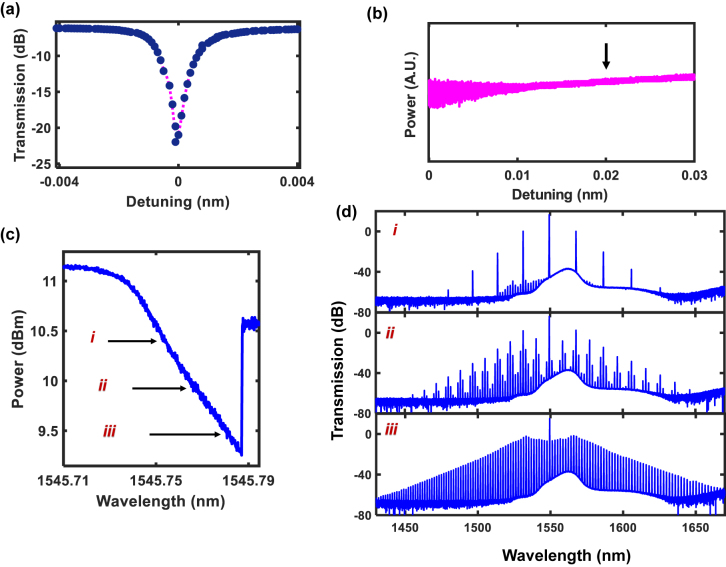
Characterization of different frequency comb states. (a) Measured transmission of a single resonance in the microresonator used to generate the frequency comb. (b) Measured output intensity of the frequency comb as a function of pump-resonator detuning. The arrow indicates the pump regime used in the experiments. (c) Measured transmission of the resonance as the laser wavelength is swept. The locations of the pump wavelength used to excite comb states ***i***, ***ii*** and ***iii*** are denoted. (d) Experimentally generated frequency comb states corresponding to the pump wavelengths shown in Figure 3(c).

Figure 3 further documents the generated frequency comb states. Figure 3(a) shows the transmission spectrum of a single resonance in the resonator used for the frequency comb generation. The frequency comb states shown were generated by sweeping a continuous-wave laser from the blue side into a preset wavelength within the resonance. Figure 3(c) shows the measured resonance when the pump is scanned into the resonance. These different preset pump wavelengths denoted in Figure 3(c) give rise to the different comb states as shown in Figure 3(d). Figure 3(b) shows the experimentally measured comb output as a function of the pump-resonance wavelength detuning. It is observed that at the detuning used in our high-speed experiments (indicated by the black arrow), the transmitted power has minimal fluctuations, and does not experience abrupt changes in amplitude when the detuning value is either increased or decreased. This is of profound significance, as it implies that the amount of intracavity power circulating in the microresonator is not subject to large fluctuations, rendering the comb state less susceptible to destabilization. Conversely, Figure 3(b) shows that when the pump-resonance detuning is decreased beyond 0.01 nm, the transmitted power fluctuates considerably with marginal wavelength changes, and is likewise not ideal for a stable output amplitude that is intrinsically required for low IMDD bit error rates. Comb state ***i*** shown in Figure 3(d) represents the pump regime for the primary comb state which we adopt for our high-speed data experiments. In modulation schemes compatible with IMDD such as NRZ and PAM4, the optical carrier undergoes intensity modulation, with NRZ and PAM4 possessing two and four different amplitude levels respectively. It is thus imperative that the output from the comb lines need to have a stable amplitude as a function of time. Comb state ***i*** was stable with minimal intensity fluctuations, important for robust modulation of IMDD data and did not require active thermal stabilization. We note further that alternate comb states in the modulation instability regime (***ii*** and ***iii***) did not yield low bit error rates or open eye diagrams, consistent with the associated intensity fluctuations previously reported in such comb states [5, 6].

**Figure 4:**
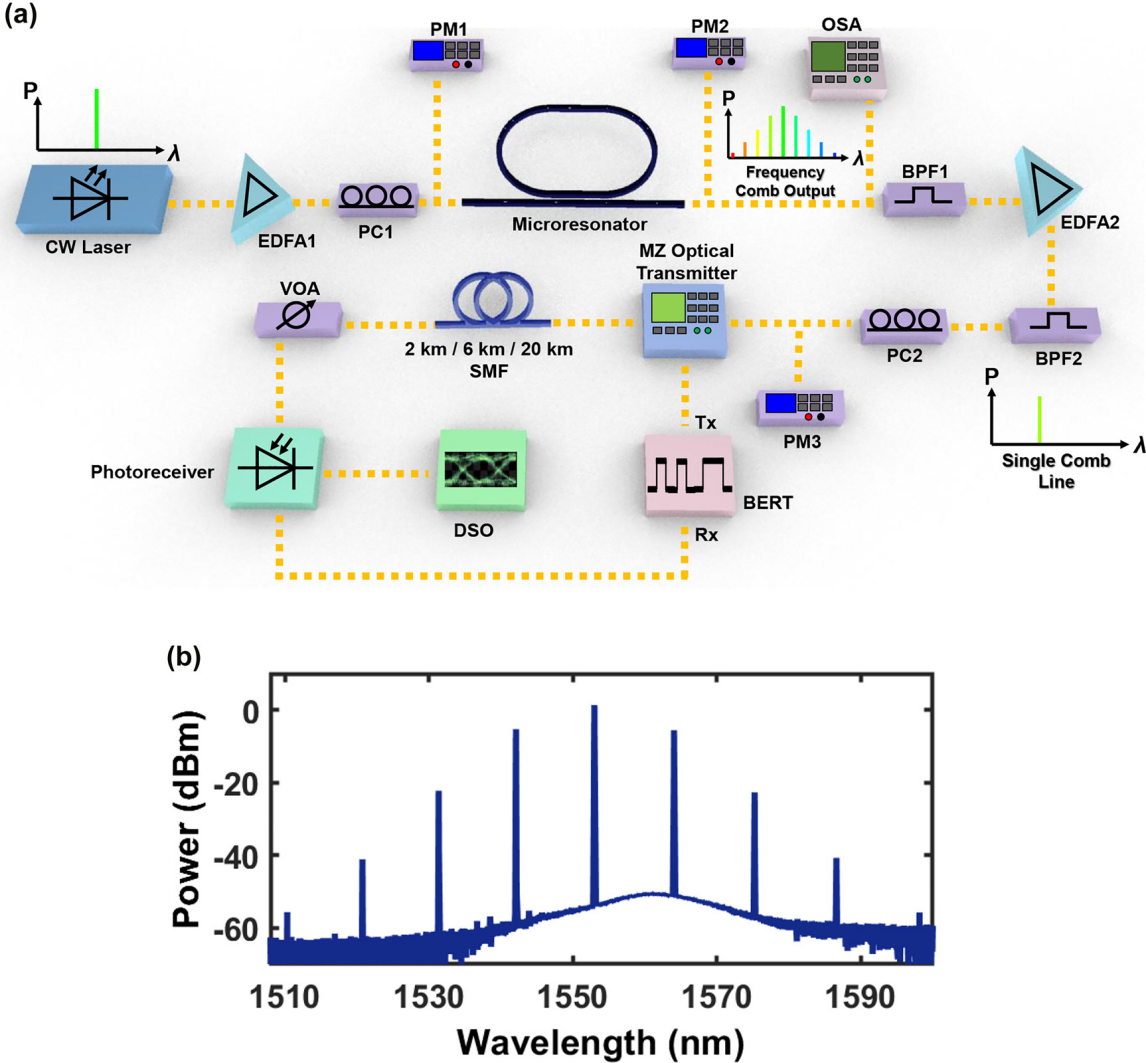
Experimental setup and frequency comb state used for the high-speed data experiments. (a) Schematic of the IMDD high-speed data experiments using the microresonator frequency comb. (b) Spectrum of the comb state used for the high-speed data experiments. The pump wavelength and power are 1553 nm and 20 mW respectively. EDFA – Erbium Doped Fiber Amplifier, PC – Polarization Controller, PM – Power Meter, BPF – Bandpass Filter, OSA – Optical Spectrum Analyzer, MZ – Mach Zehnder Transmitter, DSO – Digital Sampling Oscilloscope, BERT – Bit Error Rate Tester, Tx (Rx) – Transmit (Receive) port, VOA – Variable Optical Attenuator.

Next, we perform IMDD-compatible modulation onto the generated frequency combs. Figure 4(a) shows the schematic of the experimental setup used for the high-speed experiments. Details of the high-speed characterization are provided in Supplementary Material section. A pump at 1553 nm with a coupled power of 20 mW is used to generate the frequency comb shown in Figure 4(b). We characterize the next nearest neighbor comb lines located at 1542 nm, 1564 nm and 1575 nm. The generated comb lines are then encoded with 4 IMDD-based modulation formats: (1) 10 Gb s^−1^ NRZ, (2) 30 Gb s^−1^ NRZ, (3) 42 Gb s^−1^ PAM4, and (4) 60 Gb s^−1^ PAM4. The high-speed data is then transmitted through standard single mode fiber of lengths 2 km, 6 km and 20 km. We note that the 2 km fiber length is especially industry relevant, given that PSM4 specifications point to a maximum reach of 500 m and CWDM4 specifications point to a 2 km maximum reach [23, 25]. In addition, the 20 km optical fiber length exceeds the 10 km fiber reach stipulated by IEEE 802.3ba 100GBASE-LR4 products [27]. We first perform the high-speed characterization using NRZ data. Figure 4 shows the measured bit error rate (BER) plots for the comb lines at 1542 nm, 1564 nm and 1575 nm, which we refer to subsequently as Comb1, Comb2 and Comb3 respectively. The black, blue and red plots represent the BER plots obtained after transmission using 2 km, 6 km and 20 km of optical fiber respectively. We note that the requirements for the Received Optical Power (ROP) varies with the modulation format used. In general, higher line rates and higher bits per symbol (PAM4: 2 bits per symbol, NRZ: 1 bit per symbol) will require a higher ROP, in line with a higher required optical signal-to-noise ratio. This explains why certain experimental configurations required an additional EDFA2. Details on which configurations required EDFA2 are provided in Supplementary Material. Since our photoreceiver is PIN based, 10 Gb s^−1^ NRZ required an ROP of −20 dBm to be demodulated and a 30 Gb s^−1^ NRZ required an ROP of −15 dBm. For PAM4 data, the required ROP is about −12 dBm or higher. This information is important as designers would need to take into account the ROP requirement at the receiver end.

**Figure 5:**
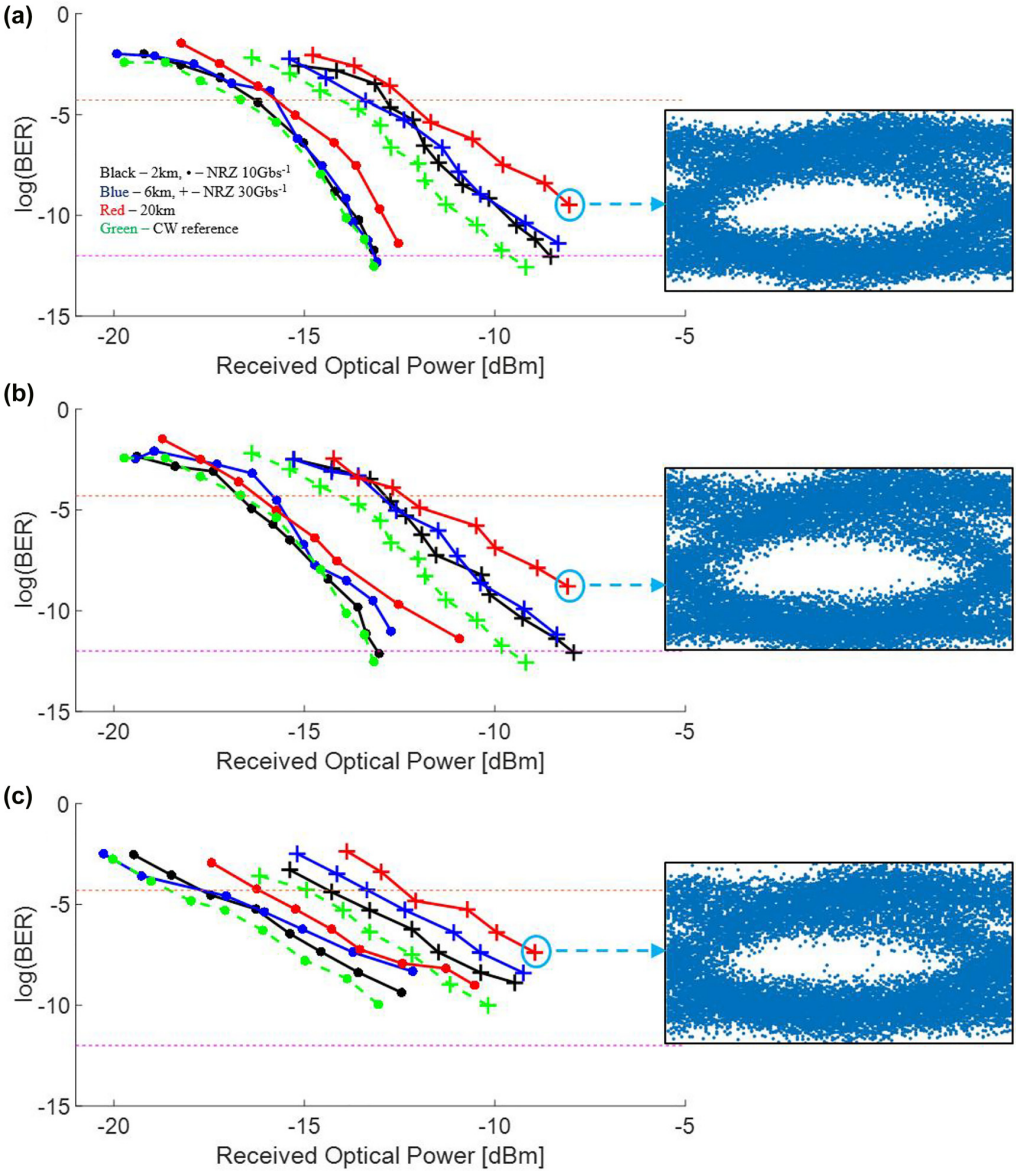
High-speed data experimental characterization using NRZ data, showing BER less than the FEC threshold and open eye diagrams for all comb lines. 10 Gb s^−1^ (**●**) and 30 Gb s^−1^ (**+**) NRZ data are modulated onto the frequency combs lines prior to transmission through 2 km (black), 6 km (blue) and 20 km (red) optical fiber. Measured bit error rate for the comb lines at (a) 1542 nm (Comb1), (b) 1564 nm (Comb2) and (c) 1575 nm (Comb3). The CW reference is shown in green for both 10 Gb s^−1^ and 30 Gb s^−1^ NRZ data. The orange and Fuchsia dotted lines denote the FEC BER limit of 5 × 10^−5^ and error free BER limit of 10^−12^ respectively. Bit error rates ≈10^−12^ well below the FEC threshold is achieved for all fiber lengths and data rates tested for Comb1 (1542 nm) and Comb2 (1564 nm). Eye diagrams for 30 Gb s^−1^ NRZ data after transmission through 20 km of optical fiber are also shown for each comb line.

The BER plots for Comb1 and Comb2 are observed from Figure 5 (a) and (b) to be largely similar. These 2 comb lines are the next nearest neighbor to the pump line. For 10 Gb s^−1^ NRZ data, we obtain a BER of between 10^−12^ to 10^−11^ at an ROP range of −11 dBm to −13 dBm even at a long fiber distance of 20 km. This is because the bit duration of 10 Gb s^−1^ data is 100 ps wide and this allows a large tolerance against fiber dispersion even at long distances.

At an NRZ data rate of 30 Gb s^−1^, each comb line is observed to have similar BER versus ROP characteristics for both 2 km and 6 km fiber lengths. This is because the incremental fiber loss and dispersion is marginal. However, at a fiber length of 20 km, the measured BER plots are observed to incur a slight increase in power penalty of about 1 dB at a BER level of 10^−9^. Firstly, 30 Gb s^−1^ data possesses a bit duration that is a third that of 10 Gb s^−1^ data. Secondly, 20 km optical fiber possesses a large aggregate dispersion of 320 ps nm^−1^ (at 1550 nm) that impairs IMDD data. These two factors contribute to the increased BER for 30 Gb s^−1^ NRZ data for 20 km optical fiber, with dispersion contributing more extensively to the degradation: We were able to obtain a bit error rate ≈10^−12^ in 2 km and 6 km fiber for both 10 Gb s^−1^ and 30 Gb s^−1^ NRZ data, but we could only obtain the smallest BER of 10^−9^ for 20 km optical fiber for Comb1 and Comb2.

**Figure 6:**
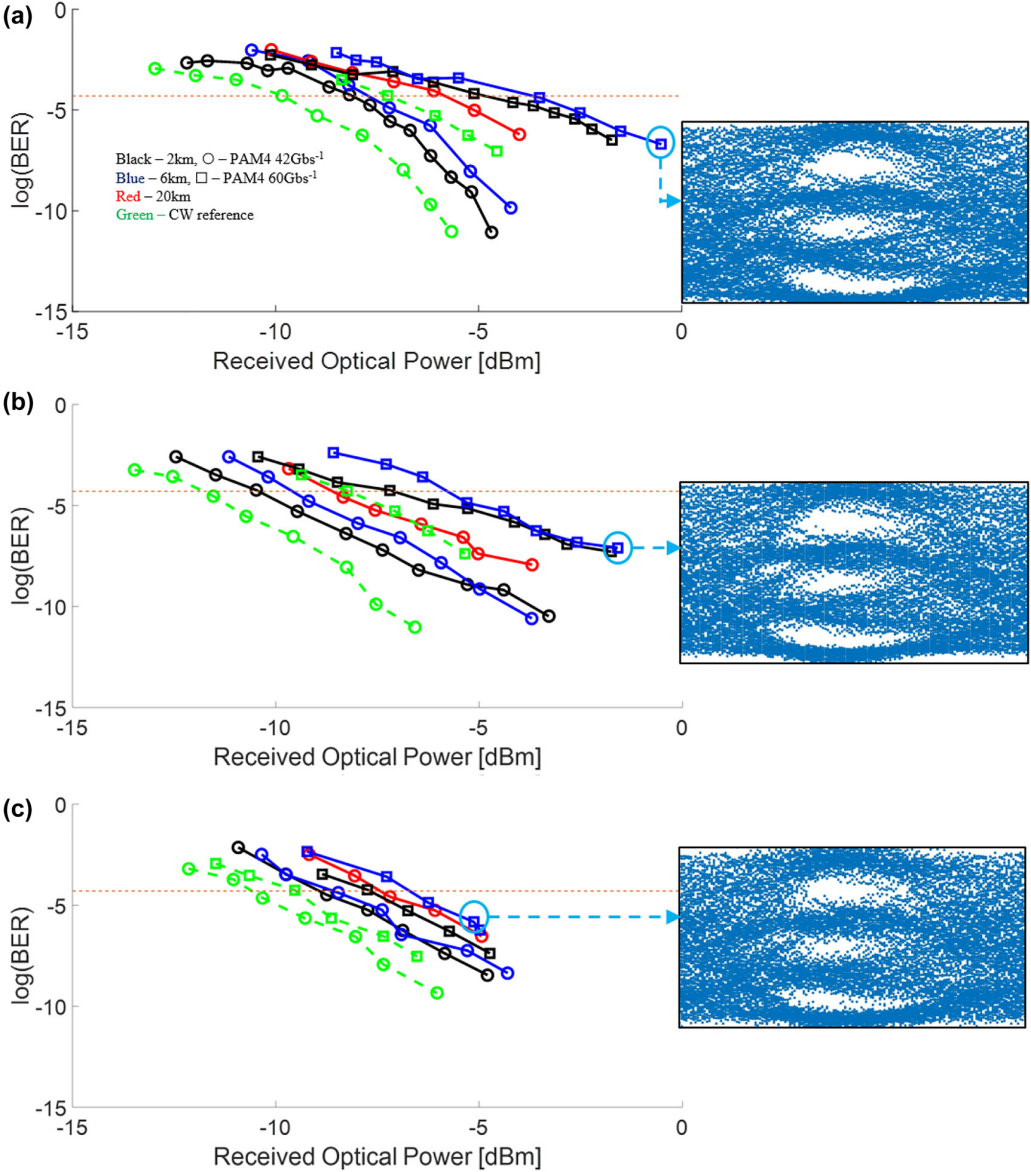
High-speed data experimental characterization using PAM4 data, showing BER less than the FEC threshold and open eye diagrams for all comb lines. 42 Gb s^−1^ (○) and 60 Gb s^−1^ (**□**) PAM4 data are modulated onto the frequency combs lines prior to transmission through 2 km (black), 6 km (blue) and 20 km (red) optical fiber. Measured bit error rate for the comb lines at (a) 1542 nm, (b) 1564 nm and (c) 1575 nm. The CW reference is shown in green for both 42 Gb s^−1^ and 60 Gb s^−1^ PAM4 data. The orange dotted lines denote the FEC BER limit of 5 × 10^−5^. BER below the FEC limit is achieved for all data rates tested and fiber lengths of 2 km and 6 km for all three comb lines. Eye diagrams for 60 Gb s^−1^ PAM4 data after transmission through 6 km of optical fiber are shown for each comb line.

Next, we perform high-speed characterization using 42 Gb s^−1^ and 60 Gb s^−1^ PAM4 data (limited to the range of data rates available in our BERT Tx PAM4 generator). Figure 6 shows the measured BER as a function of ROP for Comb1, Comb2 and Comb3. It is observed that the ROP required for 60 Gb s^−1^ PAM4 data is larger than that for 42 Gb s^−1^ for all 3 comb lines. This is due to the greater extent of eye closure which arises from the larger bit per symbol and shorter symbol duration, which in turn requires a larger ROP for characterization. For 42 Gb s^−1^ PAM4 at 2 km and 6 km, Comb1 and Comb 2 exhibited a very low BER of 10^−11^, well below the FEC threshold of 5 × 10^−5^ [24, 25]. However, when the fiber propagation length is increased to 20 km, the minimum BER measured is increased to 10^−8^. This is due to both attenuation and dispersion related impairments from the fiber, with the latter effect being more severe. We note that BER readings for 60 Gb s^−1^ PAM4 data after propagation through 20 km of optical fiber could not be obtained for all 3 comb lines. This is because the BERT Rx was not able to obtain a Receiver lock as the demodulated signal BER was higher than the 10^−2^ threshold. Although dispersion compensation is not the key focus in this manuscript, ameliorating dispersion impairments introduced by the fiber may be implemented to overcome this issue. Importantly, we note that the high BER for 60 Gb s^−1^ PAM4 data over 20 km of optical fiber is not a reflection of the frequency comb performance but a reflection of dispersion limitations that have been reported widely in the deployment of transceiver-based communication [34, 35].

For Comb3 shown in Figure 6 (c), it is observed that the lowest BER obtained was higher than that in Comb1 and Comb2. This is because in Comb3, the output at BPF1 is smaller in comparison to Comb1 and Comb2. Since the optical transmitter that modulates the data generated by the BERT’s pattern generator has an intrinsic loss of 8 dB, amplification with EDFA2 prior to input into the optical transmitter was required. The amplification introduces ASE noise which we reduce using BPF2 albeit to a limit due to its non-ideal filter roll off. This also explains why the NRZ and PAM4 eye diagrams for Comb3 are noisier than those for Comb1 and Comb2. Nevertheless, we note that the BER obtained for Comb3 is well below the FEC threshold. With the exception of 60 Gb s^−1^ PAM4 data over 20 km of fiber, we were able to satisfy the FEC threshold for all the different scenarios for fiber lengths, modulation format, line rate, and comb output power in our experiments. In terms of achieving error free rate performance (BER ≤ 10^−12^), we have successfully demonstrated this for fiber lengths of up to 6 km for 30 Gb s^−1^ NRZ data. Error free indicates that FEC implementation can be waived. In terms of the highest line rate, we have demonstrated 60 Gb s^−1^ PAM4 over 6 km fiber length with a BER of 10^−6^, well within the FEC threshold. In terms of the bitrate-distance product, which is a barometer indicating the transmission capacity in optical fiber links, we have demonstrated a single lane rate of 42 Gb s^−1^ PAM4 over 20 km of fiber with a BER of 10^−8^.

## 3 Discussion

We distinguish our work from prior work in high-speed data transmission over fiber using microresonator frequency combs, by demonstrating direct detect, intensity modulated data at data rates and reaches exceeding those in commercially deployed data center transceiver products, as opposed to coherent modulation formats and coherent detection, thus firmly cementing the applicability of frequency comb-based light sources as a promising alternative to the status quo which utilizes one laser per wavelength channel of data. We note that the comb state used for the high-speed data transmission did not require any active stabilization, and the comb output was stable because the pump-resonator detuning used, (i) resided in the regime where intensity fluctuations were absent in the comb lines and (ii) was in a regime of good thermal (wavelength) equilibrium. The output amplitude profile corresponding to the lines of specific comb states is another important determinant as to whether IMDD data may successfully use these generated wavelengths as a transmission vessel. DKS combs have been documented to temporally output different numbers of solitons, with the repetition rate of the solitons being related to the free-spectral range of the microresonator. Similar mechanisms also exist in soliton crystal states, with a taxonomy of soliton crystal states describing the frequency response of the generated comb and the corresponding number of solitons having previously been documented [28]. Considering the reliance of IMDD data on a constant, non-fluctuating output amplitude, soliton-states in high-speed data transmission are best used when the CW components in each comb line are filtered for use as individual data carriers, as has been done remarkably in Refs [20, 21]. In the context of wavelength multiplexed IMDD-based transceivers in the data center industry, wavelength spacing however needs to be much wider (on the order of 3 THz for CWDM) than the typical line spacing on the order of tens to hundreds of GHz in state-of-the-art soliton microresonator frequency combs. In our work, the comb spacing achieved was 1.4 THz, which is very close to the requisite wavelength spacing as set out by ITU CWDM requirements adopted by CWDM4-based transceiver products. Whereas in this work where 4 comb lines were characterized, future implementation may expand the number of lines in the C- and L-bands.

Table 1 shows the key transmitter specifications for products under the CWDM4 and PSM4 MSAs along with the key results. In our work, we successfully demonstrate a microresonator-based frequency comb for the transmission of high-speed NRZ and PAM4 data at 30 Gb s^−1^ and 60 Gb s^−1^ respectively. This exceeds the baud rate used by companies delivering silicon photonics-based transceiver products: CWDM4 and PSM4 MSAs for example specify a baud rate (per lane) of 25 Gbaud [24, 25, 36], [37], [38], [39], [40], [41], [42], [43], [44], [45], [46], [47]. In addition, the side mode suppression ratio which measures the difference in amplitude between the main laser mode and any side modes is specified as 30 dB for both PSM4 and CWDM4. In our frequency comb source, the side mode suppression ratio for each of the characterized frequency comb lines exceeds 35 dB. In addition to Table 1, we compare the merits of our work to recent state of the art demonstrations of frequency comb-based data transmission in Table 2, where it may be seen that frequency-comb based IMDD data transmission over fiber have largely been focused on OOK (on-off keying) and NRZ modulation at rates ≤10 Gb s^−1^. It may be noted that our work demonstrates one of the highest IMDD lane rates transmitted using a microresonator frequency comb over fiber to date.

**Table 1:** Comparison of the frequency comb source for IMDD high-speed data, with transmitter specifications for the PSM4 and CWDM4 MSAs. The metrics achieved in our frequency comb source meet or exceed those stipulated by the MSAs.

	PSM4	CWDM4	This Work	This Work	This Work
			(Longest Reach)	(Highest Bitrate)	(Smallest BER)
Reach	≤500 m	2 m–2 km	**20 km**	6 km	2 km
Single lane data rate	25 GBaud	25 GBaud	42 Gbs^−1^ PAM4	**60 Gbs** ^**−1**^ **PAM4**	30 Gbs^−1^ NRZ
BER requirement/Achieved	5 × 10^−5^	5 × 10^−5^	10^−8^	10^−6^	**<10** ^**−12**^ **(error free)**
Side mode suppression ratio (Min)	30 dB	30 dB	>35 dB	>35 dB	>35 dB

The bold values in Table 1 mean the best possible results obtained for that particular category. It is meant to emphasize the saliant features of the work.

**Table 2:** Comparison of our work against the state-of-the-art for IMDD-compatible, microresonator-based high-speed data transmission.

Reference	Highest bit rate performed	Longest fiber reach used
[37]	10 Gb s^−1^ OOK	N.A.
[38]	10 Gb s^−1^ NRZ	10 km
[39]	6 Gb s^−1^ OOK	N.A.
[40]	2 Gb s^−1^ PAM4	N.A.
[41]	25 Gb s^−1^ OOK	N.A.
[42]	10 Gb s^−1^ NRZ	40 km
[43]	10 Gb s^−1^ NRZ	N.A.
This work	60 Gb s^−1^ PAM4	6 km
This work	42 Gb s^−1^ PAM4	20 km
This work	30 Gb s^−1^ NRZ	20 km

We note further that IMDD based transceiver products typically adopt the O-band. A key reason being that chromatic dispersion in single-mode fiber is lowest at 1310 nm, and the 4 CWDM channels as specified by the international telecommunications union (1271 nm, 1291 nm, 1311 nm and 1331 nm) have substantially smaller dispersion than at the C- and L-bands [48]. The limitations imposed by dispersion impairments have been highlighted by various companies [49–52]. While coherent detectors can ameliorate impairments from dispersion, transceivers which adopt coherent detection technologies are typically only deployed at long reaches and data rates on the order of hundreds of Gb s^−1^, primarily because the associated cost, complexity and latency overheads are prohibitive for short to mid-haul transmission reaches [34–36]. While our frequency comb technology has been demonstrated at the C- and L-bands, it may easily be translated to the O-band by tailoring the pump wavelength and/or dispersion profile of the resonator for that wavelength. Despite the higher optical fiber dispersion at the wavelengths used in this work (16 ps nm^−1^ at 1550 nm vs. close to 0 ps nm^−1^ at 1310 nm), we note that the quality of the received IMDD data showed low bit error rates (below the FEC limit of 5 × 10^−5^ for all fiber lengths and data rates tested). This further illustrates that within the fiber lengths tested in this work, which far exceed the maximum reach specified by the CWDM4 MSA (2 m–2 km) and PSM4 MSA (2 m–500 m), the microresonator frequency comb demonstrated allows direct detection high-speed data to be successfully transmitted at baud rates which exceed current industry standards. We envision that in the future, the use of CMOS-compatible, homogeneously integrated on-chip dispersion compensation devices [53, 54] could avail far longer reaches without dispersion impairments, using the same comb state while enabling a monotonic left-ward shift in the bit error rate plot shown in Figure 3(a). Other approaches to overcome chromatic dispersion include digital compensation at the transmitter, digital signal processing and advanced filtering, all of which have been studied using direct modulated laser sources [55–58]. Extending their use to microresonator frequency comb-based data transmission could be another pertinent area to study, potentially enhancing the performance of frequency comb based IMDD data transmission. This work demonstrates the strong potential for on-chip, CMOS-compatible microresonator frequency combs to advance IMDD transceiver architectures in the data center industry, which today use one laser per channel (off-chip or heterogeneously integrated), importantly at commercially relevant reaches and data rates.

## Supplementary Material

Supplementary Material Details
