# Supplementary material for: Microresonator frequency comb based high-speed transmission of intensity modulated direct detection data
Source: Nanophotonics. 2022 Jun 16;11(14):3269–80. doi: 10.1515/nanoph-2022-0134 (PMC11502088; doi:10.1515/nanoph-2022-0134)
Supplement: Supplementary file 1 — Supplementary Material Details [file j_nanoph-2022-0134_suppl.docx]

Supplementary Information

Microresonator Design

The frequency comb was generated using a silicon nitride ring resonator with a radius of 100 μm and cross section of 800 nm by 1.5 μm with SiO_2_ over- and under-cladding. The footprint of the device is approximately only 0.04mm^2^. The ring’s free-spectral-range, loaded and intrinsic quality factors were of 230 GHz, 1.2 million and 1.6 million respectively. Inverse tapers were to facilitate fiber-waveguide coupling with a coupling loss of 3 dB per facet. *D*_int_ was calculated using the resonance locations of the measured microresonator transmission spectrum using the equation, <inline>{D_{\spmathit{{int}}}} = \frac{{{D_{2}}}}{{2!}}{\mu ^{2}} + \frac{{{D_{3}}}}{{3!}}{\mu ^{3}} + \cdots </inline>
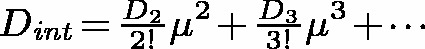
 where *μ* is the relative mode number (*μ* = 0 at the pump wavelength), *D*_2_ and *D*_3_ are the 2nd and 3rd order dispersion parameter.

Details of frequency comb generation

The transmission spectrum of the microresonators was characterized using a wavelength swept, tunable continuous-wave laser and a synchronized photodetector. The light was first adjusted for transverse-electric (TE) polarization prior to coupling into the microresonator using a lensed fiber. The output intensity of the generated frequency comb as a function of pump-resonance detuning was measured using a fiber-coupled ultrafast photodetector. To generate the microresonator frequency comb state, the tunable laser was swept from the blue side of the resonance to the specified laser-resonance detuning wavelength, at a scan rate of 10 nm s^−1^. An erbium doped fiber amplifier was used to amplify the tunable laser output. The light was adjusted for transverse-electric polarization before being coupled into the microresonator. No active thermal stabilization or temperature control was required during the frequency comb generation and high-speed experiments. The pump power used to generate the three different comb states illustrated in [Figure 3](#fig3) (d) was 100 mW, whereas the pump power used to generate the Turing pattern used for the high-speed data experiments (shown in [Figure 4](#fig4) (b)) was 20 mW.

High-speed data experiments

High-speed data was encoded onto the individual comb lines generated in the microresonator. First, with the help of a software control, the pump wavelength was swept from the blue side into the resonance to achieve Turing state (region ***i*** of [Figure 2](#fig2) (c)). The output is fed into EDFA1 and into the Fiber Polarization Controller (PC1). PC1 was used to optimize the TE configuration before launching into the resonator using a tapered fiber. 1% Power Meter taps (PM1,2) were used before and after the resonator to monitor any misalignment live during the experiments. Once the Turing microcombs were achieved as viewed on the Optical Spectrum Analyzer (OSA), a Bandpass Filter (BPF1) was used to filter the desired comb line (Comb1 or 2 or 3). The band-edge slope of BPF1 is 400dB/nm. This is important as only the desired comb line is being extracted. Its bandwidth was tuned to about 1nm. For certain configurations, the filtered comb line was amplified at EDFA2. Scenarios where EDFA2 needed to be used is shown in [Table 2](#tbl2) below. Optimizing the comb power allowed us to omit EDFA2 for 10 Gb s^−1^ NRZ in 2km and 6km fiber reaches for Comb1 and Comb2, where there was sufficient power for demodulation at the Photoreceiver. In the experiments where EDFA2 was used, BPF2 was used to eliminate the ASE noise arising from EDFA2. For the case of Comb3 located at 1575 nm, a suitable L-band EDFA was used. PC2 was used to optimize the linear polarization along the slow axis. PM3 is used for power monitoring purpose to prevent damage to the optical transmitter. The bandwidth of MZ and DSO were 35GHz and 32GHz respectively and is sufficient for the highest Baud rate used.

For high-speed characterization, a 2^31^-1 Pseudo Random Binary Sequence were used to modulate the filtered microcomb using a Mach-Zehnder Optical Transmitter. The is transmitted through a 2 km, 6 km or 20 km single mode fiber to mimic intra- and inter-data center communications of similar distances at the various modulation formats and Baud rates specified. The comb spectra were characterized one line at one time. The photoreceiver converts the optical data signal back to electrical for BER and eye diagram characterization and subsequently processed offline.

| <!--Col Count:5-->Fiber Length | NRZ 10 Gb s^−1^ | NRZ 30 Gb s^−1^ | PAM4 42 Gb s^−1^ | PAM4 60 Gb s^−1^ |
| --- | --- | --- | --- | --- |
| **2 km** | Comb3 | Combs 1, 2, 3 | Combs 1, 2, 3 | Combs 1, 2, 3 |
| **6 km** | Comb3 | Combs 1, 2, 3 | Combs 1, 2, 3 | Combs 1, 2, 3 |
| **20 km** | Combs 1, 2, 3 | Combs 1, 2, 3 | Combs 1, 2, 3 | Combs 1, 2, 3 |

Table 2: High-speed experimental configurations where EDFA2 and BPF2 were used.
